# Standard anterior peritomy versus a small posterior incision for the implantation of the PRESERFLO microshunt

**DOI:** 10.1007/s10792-023-02910-z

**Published:** 2023-10-24

**Authors:** Ahmed Bamousa, Mohamad Dakroub, Raoul Verma-Fuehring, Kosmas Papadopoulos, Jost Hillenkamp, N. A. Loewen

**Affiliations:** 1 Department of Ophthalmology, University of Würzburg, Würzburg, Germany; 2 Artemis Eye Centers of Frankfurt, Hanauer Landstr. 147, 60314 Frankfurt, Germany

**Keywords:** Glaucoma surgery, PRESERFLO microshunt, Surgical technique

## Abstract

**Purpose:**

To compare two approaches for the implantation of the PRESERFLO microshunt: an anterior approach (A) with a 6–8-mm peritomy and a posterior approach (P) with a 3-mm incision.

**Methods:**

We retrospectively analyzed 126 patients who received a PRESERFLO microshunt. We compared intraocular pressure (IOP), surgical time, medication count, and postoperative complications over nine months.

**Results:**

The baseline IOP was similar in A (21.8 ± 8.5 mm Hg) and P (23.9 ± 8.1 mm Hg) (*p* = 0.08). Surgical duration was significantly shorter in P (10 ± 0.4 min) than in A (26 ± 0.8 min) (*p* < 0.001). Postoperative IOP levels were comparable in A (10.8 ± 5.9 mm Hg) and P (10.6 ± 4.5 mm Hg) at 30 days (*p* = 0.62) and throughout the study (all intra-group *p*-values > 0.08). The preoperative medication count was 3.2 ± 1.3 drops in A and 3.3 ± 1.0 drops in P (*p* = 0.4). Postoperative values were 0.2 ± 0.6 in A and 0.3 ± 0.7 in P at nine months. There were no significant differences in complications and surgical revisions between groups (*p*-values > 0.05).

**Conclusion:**

Both techniques achieved satisfactory IOP and medication count reductions and had similar safety profiles, but the posterior incision technique was 2.6 times faster than the anterior incision technique.

**Supplementary Information:**

The online version contains supplementary material available at 10.1007/s10792-023-02910-z.

## Introduction

Traditional filtering glaucoma surgery drains aqueous humor into the subconjunctival space. It effectively lowers intraocular pressure (IOP) but has a relatively high complication rate. Ten percent of patients who undergo trabeculectomy experience intraoperative and 57% postoperative complications during the first year [1]. Various implants have been developed to drain aqueous humor into the subconjunctival or sub-tenon space with reduced surgery time, increased safety, and patient comfort. Currently, there are several tube-plate implants (tube shunts) available [2]. There are also various plateless implants that are bleb-forming. For example, the EX-PRESS (Alcon Laboratories, Inc., Texas, USA) is a medical-grade stainless steel (316LVM) device that modifies trabeculectomy [3]. The Xen gel stent (Allergan Inc., California, USA) is a hydrophilic tube made of porcine gelatin cross-linked with glutaraldehyde [4] implanted via an ab interno approach. The PRESERFLO microshunt (Santen, Osaka, Japan) is composed of a novel synthetic, thermoplastic, elastomeric biomaterial called polystyrene-block-isobutylene block-styrene (SIBS) [5].

It is implanted ab externo in an approach similar to traditional trabeculectomy, where a bleb is created using a peritomy and a fornix-based conjunctival flap. The challenge with all these devices is to balance IOP-lowering efficacy with the risk of overfiltration. Additionally, most implant materials will cause a foreign body reaction with varying degrees of encapsulation and fibrosis. In contrast to the silicone of tube shunts or the glutaraldehyde-fixated gelatin of the Xen, SIBS appears to largely avoid a foreign body reaction [6, 7] and provides a relatively low revision and needling rate [8].

The standard technique for implanting the PRESERFLO microshunt requires a large 6–8-mm peritomy along the superior cornea, which can cause bleeding, inflammation, scarring, and leakage. In this study, we propose a novel technique that uses a small 2–3-mm snip incision posterior to the limbus, which may simplify the procedure and reduce the risk of complications.

The aim of this study was to compare the efficacy and safety of the standard anterior approach and the posterior small incision approach for implanting the PRESERFLO microshunt in patients with glaucoma. Our research question was: Does the posterior small incision approach offer any advantages over the standard anterior approach in terms of IOP reduction, medication count reduction, surgical time, and postoperative complications?

To answer this question, we conducted a retrospective analysis of 126 patients who received a PRESERFLO microshunt using either technique. We measured and compared their IOP, medication count, surgical time, and postoperative complications over nine months.

## Methods

This study was a retrospective analysis of the charts of 126 glaucoma patients who underwent a PRESERFLO implantation between September 2020 and July 2021 at the University Hospital of Würzburg. The institutional review board (IRB) of the hospital granted an exemption from a formal review for this study. We followed the principles stated in the Declaration of Helsinki. We categorized the patients according to the implantation site of the PRESERFLO microshunt and divided them into an anterior group (A) and a posterior group (P). The surgical indication was uncontrolled IOP, intolerance to glaucoma drops, or clinically significant glaucomatous progression. We defined progression as a statistically significant decline of the retinal nerve fiber layer measured by Heidelberg OCT. Individuals younger than 20 and those with neovascular or trauma-induced glaucoma were excluded. Demographic parameters such as age, gender, glaucoma type, implantation site, baseline mean deviation of the visual field as a criterion for glaucoma severity, concomitant cataract surgery, and systemic diseases (e.g., diabetes) were recorded. The patients were asked to present for follow-up visits at 1, 5, 30, 90, 180, and 270 days after surgery. The IOP, visual acuity, and medication number were measured at every follow-up visit. The positions of the microshunt in the anterior chamber and under the conjunctiva were also visually assessed at the slit lamp at every follow-up visit. The total number of revisions, postoperative injections of subconjunctival 5-fluorouracil, and other postoperative complications such as fibrosis or erosion were noted.

### Surgical technique

**Fig. 1:**
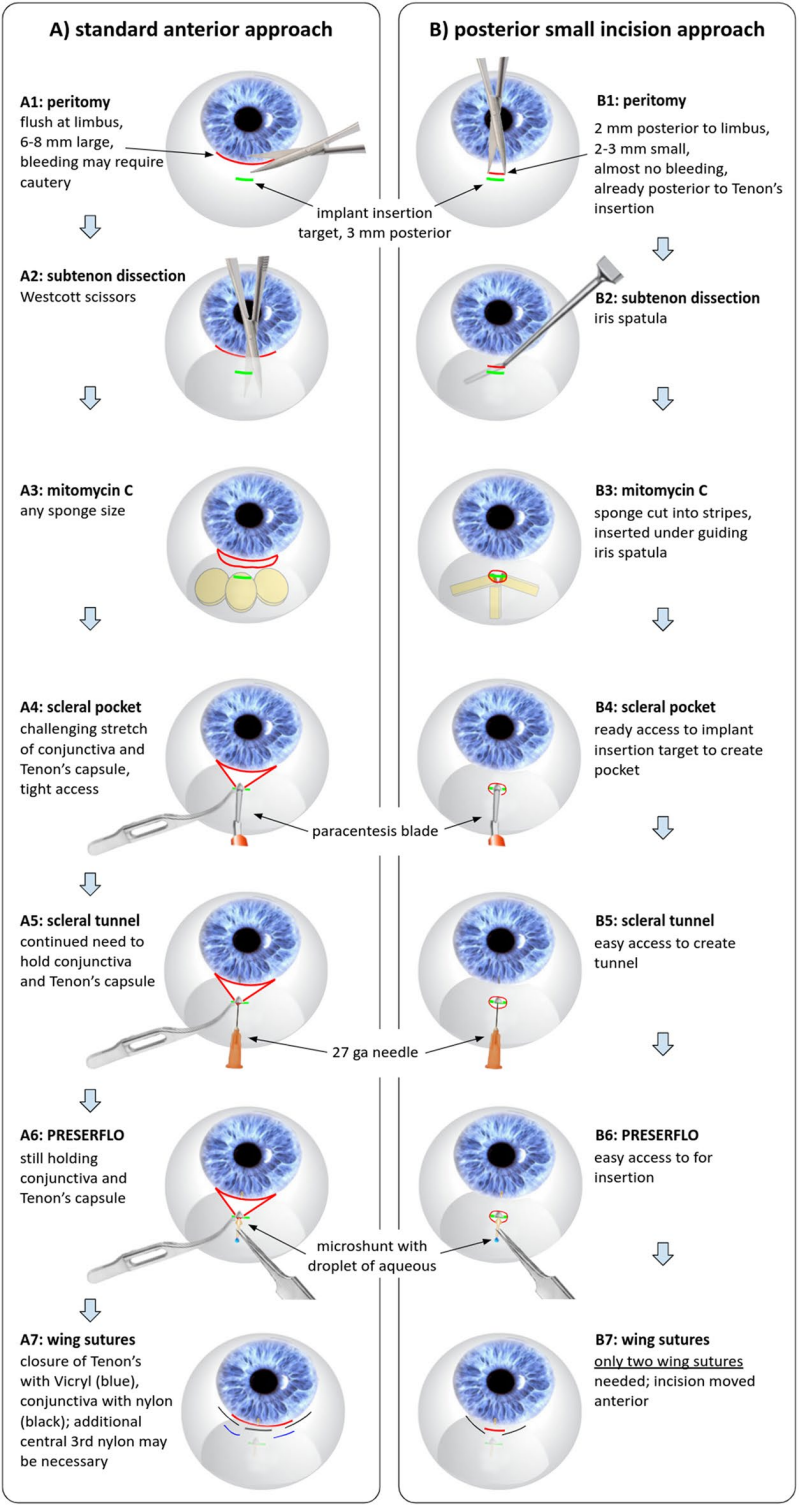
Comparison of the standard anterior (**A**) and the posterior small incision (**B**) approaches. The main difference was the size and location of the peritomy: large and anterior in A (**A1**) and small and posterior in B (**B1**). All steps in B were done through a 2–3-mm window. An iris spatula enabled effective dissection (**B2**) and insertion of mitomycin C-soaked sponges (**B3**). The incision was close to the implant insertion site, providing easy access to create the sclera pocket (**B4**) and tunnel (**B5**) and to implant the microshunt (**B6**). The small incision in B was closed with two wing sutures that moved it toward the limbus and away from the implant (**B7**)

We used two different surgical approaches (Fig. 1) performed by a surgeon experienced with the ab externo implantation of a microshunt and with small incision trabeculectomy (NAL).

### Standard, anterior approach

A superior corneal traction suture was placed approximately 1 mm anterior to the limbus. The standard anterior technique recommended by the manufacturer of the microshunt (group A) was followed. A 6–8-mm peritomy along the superior cornea was made using Westcott scissors (Fig. 1, A1). The sub-tenon space was also dissected using the same instrument (Fig. 1, A2). Three sponges soaked in mitomycin C (0.5 mg/ml) were inserted for three minutes (Fig. 1, A3), followed by copious irrigation with buffered saline solution (BSS). A 2-mm-long and 1-mm-wide pocket three millimeters posterior to the limbus was created with a paracentesis blade included in the kit (Fig. 1, A4). A 25-gauge needle was inserted into the pocket with the bevel up and advanced into the anterior chamber halfway between the cornea and the iris (Fig. 1, A5). With the bevel pointing up, the microshunt was moved into the tunnel until the wings were secured within the sclera pocket (Fig. 1, A6). The microshunt’s function was confirmed by visualizing aqueous humor exiting the tail. Tenon’s capsule and the conjunctiva were advanced to cover the microshunt and re-approximated at the limbus. Tenon’s capsule was then secured with a 7-0 polyglactin 910 suture (Vicryl, Ethicon, New Jersey, USA) and the conjunctiva with wing sutures using 10-0 nylon (Fig. 1, A7). Occasionally, a central loop stitch parallel to the limbus was placed to reduce leakage. All knots were rotated. The bleb that formed was checked for leakage. The postoperative regimen comprised dexamethasone drops four times a day for a month and then tapered by one drop per week and ofloxacin drops four times a day for a week.

### Posterior, small incision approach

We used a posterior technique with a 2–3-mm-wide peritomy and a direct opening of Tenon’s layer 2 mm posterior to the limbus (Fig. 1, B1). The sub-tenon space of the bleb was dissected with an iris spatula (Fig. 1, B2), and mitomycin C sponges were cut into a thinner but longer shape (Fig. 1, B2). The scleral pocket, tunnel, device insertion, and priming were done like in the standard implantation technique but through the conjunctival incision that readily exposed the implantation site (Fig. 1, B4-6). The closure differed from the standard technique in that the 2–3-mm peritomy was pulled over the implant and positioned directly at the limbus instead of at the original position 2 mm posterior to the limbus (Fig. 1, B7). This was done to move the incision away from the implant’s scleral pocket and to prevent the implant from touching Tenon’s capsule or the conjunctiva. Tenon’s layer and conjunctiva were secured at the limbus in a watertight fashion with a single 10–0 nylon wing suture on each side of the incision. The same postoperative regimen as in group A was used.

### Statistical analysis

We used SPSS (version 26, IBM, New York, USA) for statistical analyses. We recorded parameters as dichotomous or continuous variables. Continuous variables were reported as means and standard deviations. The Kolmogorov–Smirnov test was used to check for a normal distribution of the parameters studied. Means of normally distributed datasets were compared using t tests and those with a non-normal distribution using Mann–Whitney U tests. A Chi-square test was deployed to check for significant differences between dichotomous variables. A *p*-value of 0.05 or less was considered statistically significant for all our analyses. Surgical success was defined as a pressure reduction by at least 30% and an IOP below or equal to 14 mmHg.

## Results

We included 126 eyes in the statistical analysis: 54 eyes in group A and 72 eyes in group P. Table 1 shows the baseline demographics for A and P. The mean ages in both groups were similar: 72.3 ± 12.1 years in A and 70.2 ± 10.7 years in P (*p* = 0.29). The male-to-female gender ratio was 1:1.7 in A and 1:1.2 in P; these figures did not differ significantly (*p* = 0.32). Primary glaucomas were the most common glaucoma type in our population: 79.6% in A and 77.8% in P. The mean preoperative IOP value was 21.8 ± 8.5 mmHg in A and 23.9 ± 8.1 in P, which were not statistically different (*p* = 0.08). The number of glaucoma eye drops at baseline was also comparable in both groups: 3.2 ± 1.3 in A and 3.3 ± 1.0 in P (*p* = 0.63). Visual field tests in A and P showed a similar mean defect: 12.3 ± 9.4 dB and 10.8 ± 8.1 dB, respectively (*p* = 0.40).

**Table 1:** Baseline characteristics

Parameter/technique	Anterior (*n* = 54)	Posterior (*n* = 72)	*p*-value
Age (years) (mean ± SD)	72.3 ± 12.1	70.2 ± 10.7	0.29
Gender ratio (M:F)	1: 1.7	1: 1.2	0.32
Glaucoma type primary (n, %) *Secondary (n, %)*	43 (79.6%) 11 (20.4%)	56 (77.8%) 16 (22.2%)	0.77
Baseline IOP (mm Hg) (mean ± SD)	21.8 ± 8.5	23.9 ± 8.1	0.08
Same session phaco (n, %)	7 (13.0%)	14 (19.4%)	0.33
Visual field mean defect (dB)	12.3 ± 9.4	10.8 ± 8.1	0.40
Number of baseline medications (drops) (mean ± SD)	3.2 ± 1.3	3.3 ± 1.0	0.63

Same session phaco: surgery combined with phacoemulsification

Table 2 presents the postoperative outcomes of A and P. Both techniques resulted in a significant IOP drop from baseline at every follow-up (all *p*-values < 0.05). Postoperative IOP levels were at 10.8 ± 5.9 mmHg in A and 10.6 ± 4.5 mm Hg in P at 30 days (*p* = 0.62) and remained at a similar level (all intra-group *p*-values > 0.08). There was no significant difference in IOP levels between A and P on days 30, 90, 180, and 270 (all *p*-values > 0.05). Surgical success was achieved in 68.5% of A and 75.0% of P (*p* = 0.42).

**Table 2:** Postoperative outcomes for the anterior and posterior insertion techniques

	Anterior	Posterior	*p*-value
*IOP (mmHg) (Mean ± SD) *			
day 30 day 90 day 180 day 270	10.8 ± 5.9 (*n* = 51)	10.6 ± 4.5 (*n* = 69)	0.62
	10.4 ± 3.4 (*n* = 44)	11.5 ± 4.4 (*n* = 62)	0.31
	12.0 ± 4.3 (*n* = 12)	11.8 ± 4.2 (*n* = 44)	0.95
	10.4 ± 2.1 (*n* = 7)	10.8 ± 2.5 (*n* = 12)	0.76
Number of medications (mean ± SD)	0.2 ± 0.6 (*n* = 54)	0.3 ± 0.7 (*n* = 72)	0.19
Number of 5-fu (mean ± SD)	1.9 ± 1.7	2.7 ± 2.5	0.14
Number of revisions (n, %)	13 (10.3%)	10 (7.9%)	0.14
*Complications (n, %):*			
Conjunctival scarring	13 (10.3%)	18 (14.3%)	0.90
Hyphema	1 (1%)	0 (0%)	0.25
Choroidal effusion	9 (7.1%)	11 (8.7%)	0.83
Bleb leakage	2 (1.6%)	1 (0.8%)	0.40
Tube dislocation	1 (0.8%)	0 (0%)	0.25
Conjunctival erosion	0 (0%)	1 (0.8%)	0.39

**Fig. 2:**
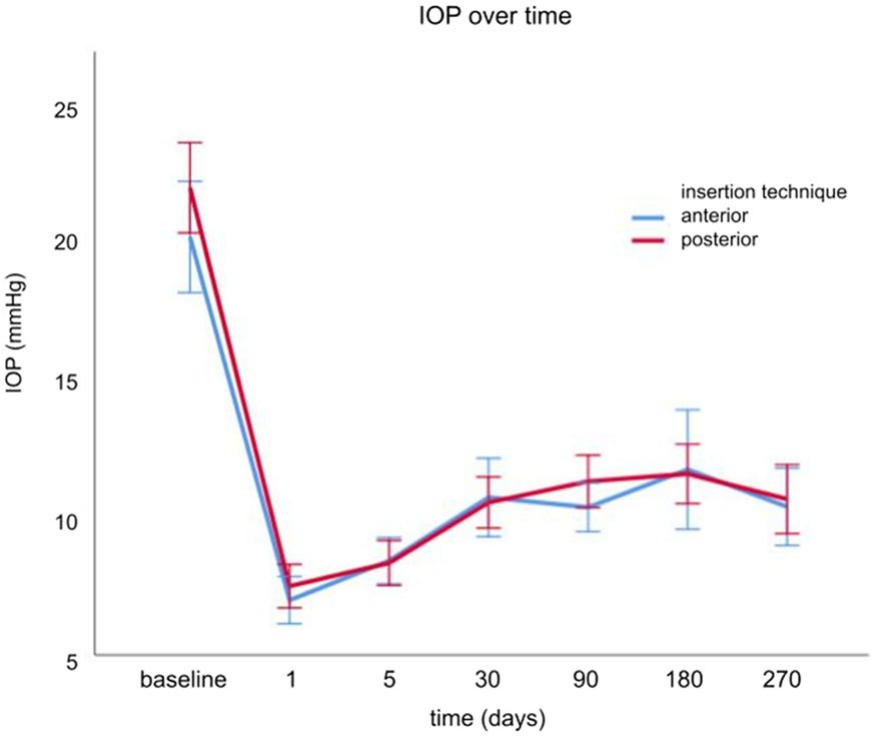
Mean IOPs before and after surgery for anterior and posterior insertion techniques. There was no significant difference in IOP levels between the two methods at any time point (*p* > 0.05)

Figure 2 illustrates the IOP curves for both techniques. The PRESERFLO significantly reduced glaucoma drops to 0.2 ± 0.6 in A and 0.3 ± 0.7 in P (both *p*-values < 0.05).

The difference between surgical techniques was most evident in the time required for each procedure: A took an average of 26 ± 0.8 min, while B took only an average of 10 ± 0.4 min (*p* < 0.001). The larger anterior incision through the vessel-rich perilimbal conjunctiva caused more bleeding and made it more difficult to visualize the target site where the implant was to be inserted (Supplementary Material 1). It was also challenging to remove mitomycin C-soaked sponges using the standard anterior technique (Supplementary Material 1, A3), which led the manufacturer of the PRESERFLO to suggest threading these sponges on a Vicryl suture for easier retrieval. To create the scleral pocket and tunnel for the implant, we had to pull back and hold under tension the conjunctiva and Tenon’s layer, which were relatively posterior to the limbus. The tension often caused the edges of the peritomy to tear, resulting in more bleeding (Supplementary Material 1, A3-6). We needed a second instrument (Colibri forceps (Fig. 1, A4-6) or stable swab (Supplementary Material 1, A4-6.2)) to stretch and hold the conjunctiva firmly in place.

These issues were avoided with the posterior small incision approach (B1). An iris spatula allowed effective dissection (B2) and insertion of mitomycin C-soaked sponges (B3). The incision was close to the implantation site, providing easy access to create the sclera pocket (B4) and tunnel (B5) and to implant the microshunt (B6). Because the incision was so small in B, we could close it easily and reliably with two wing sutures that moved the incision toward the limbus and away from the implant (B7).

During the first postoperative weeks, individuals received a similar average number of subconjunctival injections of fluorouracil-5 (50 mg/ml, 0.2 ml):1 0.9 ± 1.7 in A and 2.7 ± 2.5 in P (*p* = 0.14). In A, 13 patients (10.3%) needed a revision and 10 (7.9%) in P (*p* = 0.14). The most common postoperative complication was bleb failure from fibrosis and choroidal effusion. The number of complications for both groups was similar (all *p*-values > 0.05).

## Discussion

Eye drops can effectively treat primary open-angle glaucoma (POAG), but only 10% of patients achieve continuous treatment success one year after starting therapy with the latest prostaglandin analogs [9]. Moreover, 59% of patients experience peripheral vision loss during initial medical treatment [10], and 32% require surgery within one year [11]. Therefore, there is a need for standardized, reliable, and safe glaucoma surgery. To address this need, we developed a new surgical technique for a recently introduced glaucoma implant. We compared the standard technique, which requires a large 6 to 8-mm peritomy, with a posterior technique, which uses a small 2–3-mm snip incision. We designed the posterior technique to simplify the surgery and standardize the conjunctival closure. The standard technique had several drawbacks, such as the difficulty of accessing the sclera 3 mm posterior to the limbus, where the sclera pocket had to be created, while pulling the conjunctiva back firmly. The closure of both Tenon’s layer and the conjunctiva was also challenging.

Another concern was the unreliable watertight closure of the large peritomy, which sometimes required a loop stitch in the center of the incision that could compromise the PRESERFLO passing through the same location. These issues reduced the potential of the PRESERFLO to minimize tissue manipulation in a less invasive glaucoma surgery.

We did not find any statistically significant difference in the clinical outcomes between the two surgical techniques after nine months of follow-up. Both approaches were equally safe and effective. Common complications of PRESERFLO implantations include hypotony with or without choroidal detachment, hyphema, wound leak, tube exposure, and surgical failure leading to needling and surgical revision [12, 13]. In our study, we did not observe any differences in fibrosis, number of bleb revisions, or 5 FU injections between the two groups. One possible reason for the similar clinical outcomes is that the implantation of the microshunt was not changed. The main difference was the ease and speed of the surgery, with the posterior technique being 2.6 times faster than the standard technique (10 vs. 26 min in P and A, respectively), confirming our hypothesis. This difference may be surprising, but can be explained by how little the conjunctiva or Tenon’s capsule had to be manipulated. The small size of the incision allowed two wing sutures to pull it toward the limbus and close it securely. We also expected a lower rate of conjunctival erosion and device exposure in this group because of the smaller incision size. We did not detect any Seidel-positive leaks in either group. Previous studies have shown that longer surgery increases the risk of complications [14, 15]. Therefore, reducing surgical time should be a universal goal for surgeons, hospitals, and policymakers to avoid complications.

Few studies have evaluated the PRESERFLO due to its recent introduction and variable market approval times [5, 16–20]. Pinchuk et al. reported a case series of 23 consecutive successful microshunt implantations [7]. They observed a substantial postoperative IOP reduction and minor complications such as minimal hypotony and bleb encapsulation. They concluded that this procedure was an alternative to primary trabeculectomy. Riss et al. reported similar results in a retrospective study with a one-year follow-up [21]. They reported a 38 to 55% IOP reduction and a 72 to 85% medication reduction. Other studies confirmed these findings [5, 17–20]. Batlle et al. published 3-year results in 23 eyes [17]. The success rate for an IOP of 14 mm Hg or less and an IOP reduction of more than 20% was 100%, 91%, and 95% at 1, 2, and 3 years of follow-up, respectively [17]. The mean IOP decreased from 23.8 ± 5.3 to 10.7 ± 2.8, 11.9 ± 3.7, and 10.7 ± 3.5 mm Hg, and the mean number of glaucoma medications per patient decreased from 2.4 ± 0.9 to 0.3 ± 0.8, 0.4 ± 1.0, and 0.7 ± 1.1, respectively. The most common complications were transient hypotony (13%) and transient choroidal effusion (8.7%), which resolved spontaneously. There were no cases of leak, infection, migration, erosion, persistent corneal edema, or other serious long-term adverse events [17]. Our follow-up duration was shorter, but our results were similar.

Our study had some limitations due to its retrospective design. Other limitations were the shorter follow-up duration compared to previous studies and the visual assessment of the microshunt position and bleb morphology with a slit lamp instead of an OCT device. Gambini et al. [22] compared the bleb morphology of two different devices, the PRESERFLO and the Xen, implanted in two different anatomical locations, sub-tenon, and subconjunctival, respectively. They found significant differences in bleb characteristics, such as height, area, vascularity, and encapsulation. In our study, we implanted the PRESERFLO device sub-tenon in both groups, but used an anterior and a posterior incision. We did not assess the bleb morphology using a grading system or anterior segment OCT (AS-OCT), as we had previously shown that this has little predictive value for surgical success [23]. This is a limitation of our study that we acknowledge. However, we also caution that bleb morphology should be interpreted with care. Bleb grading can be subjective, non-standardized, and insensitive to changes over time. Moreover, AS-OCT may not capture the entire bleb or its function. Therefore, we suggest further research on the impact of bleb morphology on the outcomes of PRESERFLO implantation.

Moreover, when comparing both techniques and their surgical success, it is plausible that a larger number of patients would be needed to detect more subtle IOP differences of 1 mm Hg. Material researchers and other surgeons who provided feedback on the new technique emphasized the importance of covering the implant with both Tenon’s capsule and the conjunctiva to prevent erosion. They also advised ensuring that the wing sutures pull the incision anteriorly toward the limbus to create enough space between the scleral pocket of the implant and the conjunctiva.

## Conclusion

In conclusion, we found that the posterior insertion technique was 2.6 times faster and easier than the standard approach for implanting the PRESERFLO microshunt. Both techniques achieved comparable outcomes in terms of postoperative IOP levels, medication counts, and complications at the nine-month follow-up. Our results confirm the efficacy and safety of the PRESERFLO microshunt in reducing IOP and medication burden in patients with POAG.

### Supplementary Information

Below is the link to the electronic supplementary material.

Supplementary file1 (PNG 6516 kb)

Supplementary file2 (DOCX 13 kb)

## Data Availability

Data are available from the corresponding author on request.
