# Supplementary figures and images for: Standard anterior peritomy versus a small posterior incision for the implantation of the PRESERFLO microshunt
Source: Int Ophthalmol. 2023 Oct 24;43(12):5071–8. doi: 10.1007/s10792-023-02910-z (PMC10724329; doi:10.1007/s10792-023-02910-z)

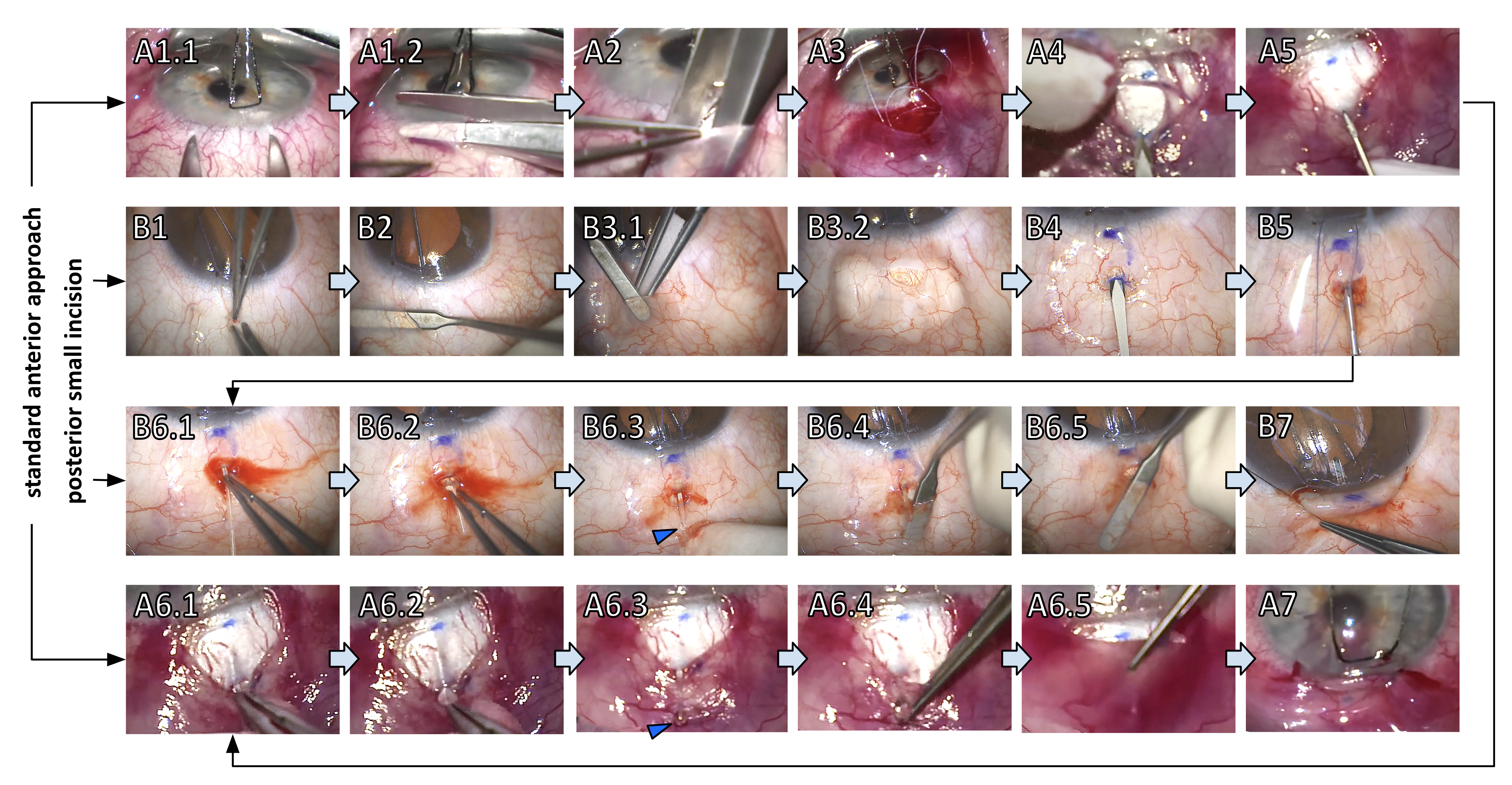

Supplement: Supplementary file 1 — Supplementary file1 (PNG 6516 kb) [file 10792_2023_2910_MOESM1_ESM.png]
