# Supplementary material for: Standard anterior peritomy versus a small posterior incision for the implantation of the PRESERFLO microshunt
Source: Int Ophthalmol. 2023 Oct 24;43(12):5071–8. doi: 10.1007/s10792-023-02910-z (PMC10724329; doi:10.1007/s10792-023-02910-z)
Supplement: Supplementary file 2 — Supplementary file2 (DOCX 13 kb) [file 10792_2023_2910_MOESM2_ESM.docx]

## Supplementary Material legends

**Supplementary material 1:** Intraoperative photographs of the standard anterior (A) and the posterior small incision (B) approaches, with corresponding steps shown side-by-side. The steps include peritomy (A1.1-1.2), bleb dissection (A2), mitomycin C sponges on a Vicryl suture (A3), scleral pocket (A4) and tunnel (A5) creation, implant insertion (A6.1-6.2), function checking (blue arrowhead in A6.3 shows aqueous exiting tail), positioning of the tail under Tenon’s and conjunctiva (A6.4-6.5) and bleb closure (A7). Similar steps are shown in B, where the surgery was done through a posterior 2-3 mm snip incision (B1) close to the implantation site. An iris spatula enabled effective dissection (B2) and insertion of mitomycin C-soaked strips of sponges (B3).
